# Supplementary figures and images for: SARS-CoV-2 Omicron variations reveal mechanisms controlling cell entry dynamics and antibody neutralization
Source: PLoS Pathog. 2024 Dec 2;20(12):e1012757. doi: 10.1371/journal.ppat.1012757 (PMC11637440; doi:10.1371/journal.ppat.1012757)

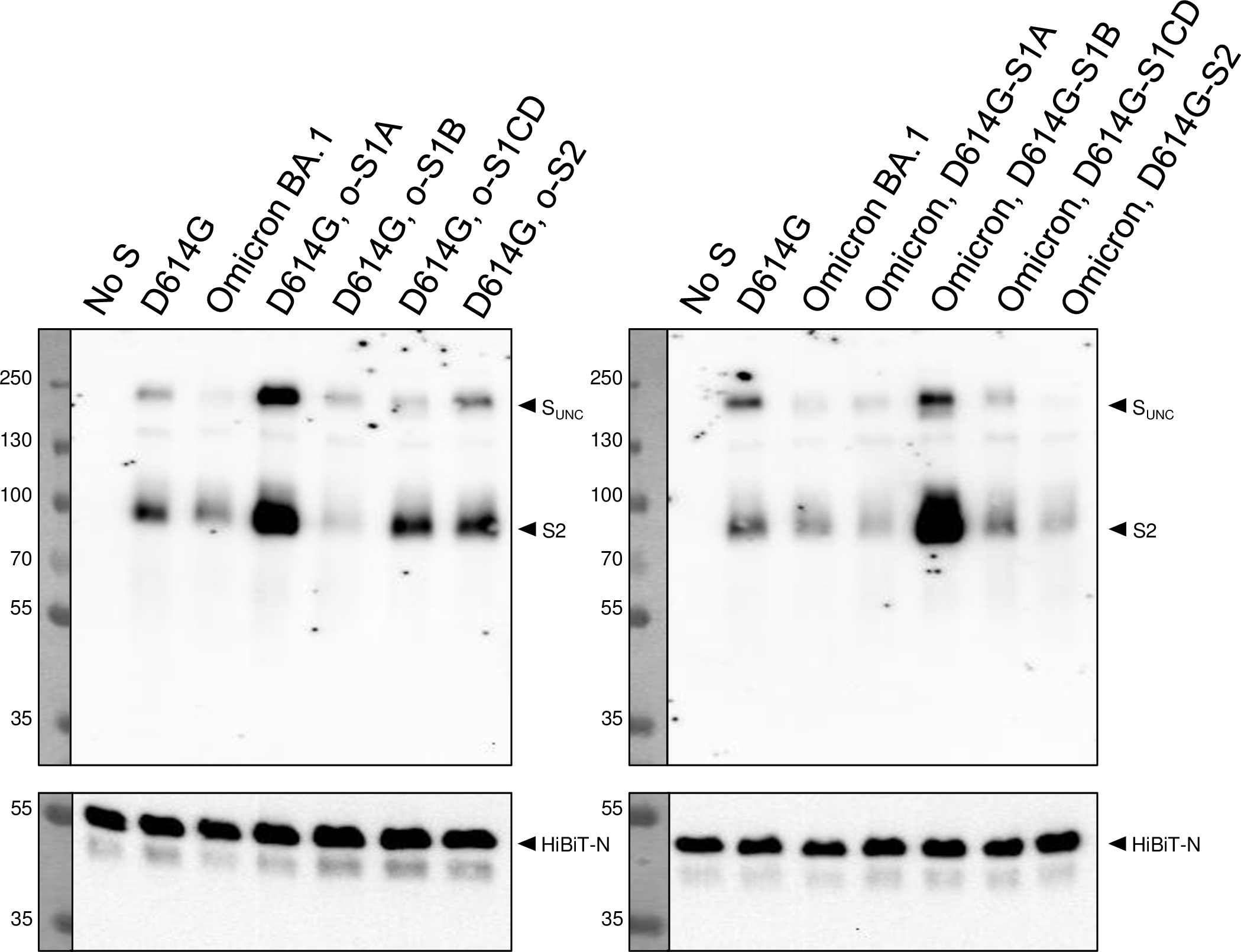

Supplement: S1 Fig — The incorporation of recombinant S proteins on VLPs. SEC-purified VLPs bearing various recombinant S proteins were subjected to WB analysis of S incorporations. SUNC, S2, S2’, and HiBiT-N are labeled. S1A or S1B domain-swapped recombinant S proteins affected S incorporation onto VLPs. This is caused by differential S maturation in producer cells, and have been found to not affect their differential sensitivity towards entry inhibitors [24]. (TIF) [file ppat.1012757.s001.tif]

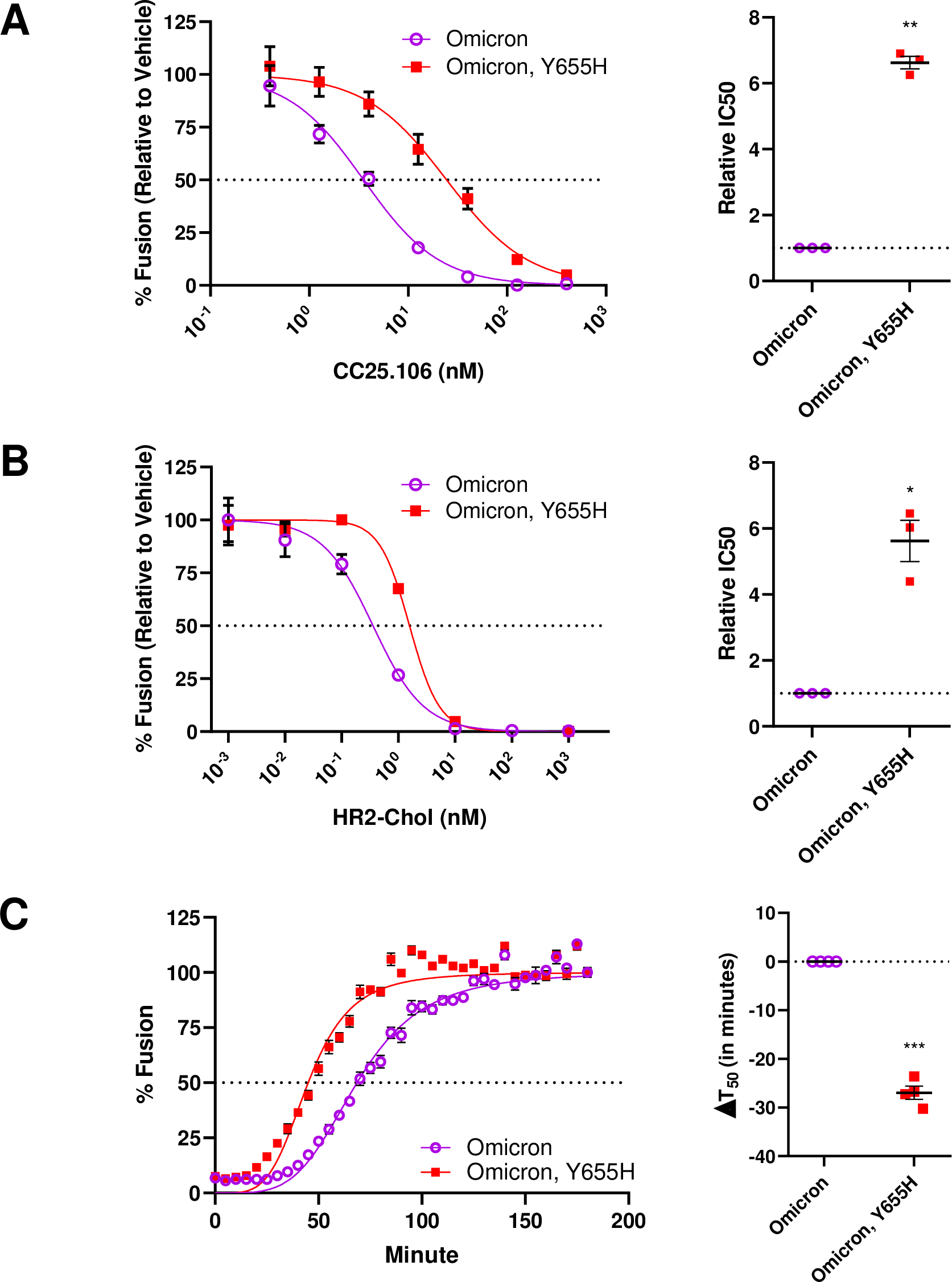

Supplement: S2 Fig — The SARS-2 VLP cell-free fusion neutralization profile of stem-helix antibody CC25.106 (A) or HR2-chol (B). Indicated concentrations of inhibitors were incubated with VLPs bearing either Omicron BA.1 or Omicron BA.1 Y655H revertant S for 30 min at 37°C before EVs, DrkBiT, substrate, and trypsin were added and RLU quantified. Left, dose-response curves between treatment and % fusion. Right, experiment on the left was repeated three times (N = 3) and the doses generating 50% inhibition (IC50) were plotted. Mean and SEM are depicted. Deviations from the reference value of 1.0 (Omicron BA.1 S) were analyzed by one-sample t tests. *, p < 0.05; **, p < 0.01. C. Cell-free membrane fusion kinetics of VLPs bearing either Omicron BA.1 or Omicron BA.1 Y655H revertant recombinant S proteins. Left, fusion kinetics curves from a representative experiment, where RLU was read every 5 min up to 180 min. Right, experiment on the left was repeated four times (N = 4) and the times generating 50% fusion (T50) were plotted. Mean and SEM are depicted. Deviations from the reference value of 0 (Omicron BA.1 S) were analyzed by one-sample t tests. ***, p < 0.001. (TIF) [file ppat.1012757.s002.tif]
