# Supplementary material for: SARS-CoV-2 Omicron variations reveal mechanisms controlling cell entry dynamics and antibody neutralization
Source: PLoS Pathog. 2024 Dec 2;20(12):e1012757. doi: 10.1371/journal.ppat.1012757 (PMC11637440; doi:10.1371/journal.ppat.1012757)
Supplement: Attachment — Submitted filename: 240128 PPATH Response to Reviews.docx [file ppat.1012757.s004.docx]

Dear dr. Gallagher,

Thank you very much for submitting your manuscript "SARS-CoV-2 Omicron variations reveal mechanisms controlling cell entry dynamics and antibody neutralization" for consideration at PLOS Pathogens. As with all papers reviewed by the journal, your manuscript was reviewed by members of the editorial board and by several independent reviewers. The reviewers appreciated the attention to an important topic. Based on the reviews, we are likely to accept this manuscript for publication, providing that you modify the manuscript according to the review recommendations.

Please prepare and submit your revised manuscript within 30 days. If you anticipate any delay, please let us know the expected resubmission date by replying to this email.

When you are ready to resubmit, please upload the following:

[1] A letter containing a detailed list of your responses to all review comments, and a description of the changes you have made in the manuscript.

Please note while forming your response, if your article is accepted, you may have the opportunity to make the peer review history publicly available. The record will include editor decision letters (with reviews) and your responses to reviewer comments. If eligible, we will contact you to opt in or out

[2] Two versions of the revised manuscript: one with either highlights or tracked changes denoting where the text has been changed; the other a clean version (uploaded as the manuscript file).

Important additional instructions are given below your reviewer comments.

Thank you again for your submission to our journal. We hope that our editorial process has been constructive so far, and we welcome your feedback at any time. Please don't hesitate to contact us if you have any questions or comments.

Sincerely,

Bart L. Haagmans

Academic Editor

PLOS Pathogens

Alexander Gorbalenya

Section Editor

PLOS Pathogens

Michael Malim

Editor-in-Chief

PLOS Pathogens

orcid.org/0000-0002-7699-2064

***********************

Reviewer Comments (if any, and for reference):

Reviewer's Responses to Questions

**Part I - Summary**
Please use this section to discuss strengths/weaknesses of study, novelty/significance, general execution and scholarship.

Reviewer #1: The manuscript entitled “SARS-CoV-2 Omicron variations reveal mechanisms controlling cell entry dynamics and antibody neutralization” by Qing and colleagues demonstrated how the H655Y substitution in the Omicron Spike protein alters viral entry kinetics and membrane fusion. Multiple studies have shown the role of the H655Y substitution in stabilizing Spike and preferential cathepsin-mediated entry of Omicron in cell lines. However, Qing and colleagues showed that the H655Y substitution alters viral fusion kinetics, potentially by prolonging the extended intermediate state of S prior to viral and target cell membrane fusion. It is understandable that such a state would increase the chance of antibodies against these epitopes to bind and prevent fusion.

Reviewer #2: The manuscript by Tang and colleagues investigates the impact of the H655Y mutation, frequently found in Omicron S1 subunits, on the membrane fusion process. This mutation delays membrane fusion by affecting a post-S2’ cleavage event, which is analyzed using a neutralizing antibody targeting the stem-helix region of S2. The author proposes a new model of SARS-CoV-2 fusion based on their findings. The experiments are generally well-executed, and the conclusions are supported by the data. That said, the manuscript would benefit from a more detailed discussion of existing studies, including comparisons between cell-cell fusion vs cell-free fusion of D614G and Omicron, and an explanation of the discrepancies in the literature regarding the action of H655Y. Additionally, confirming the results with other Omicron spike proteins would strengthen the findings. It would also be valuable to discuss whether similar mechanisms might apply to other human pathogenic coronaviruses, particularly SARS-CoV.

Reviewer #3: This is an excellent study that presents a modified model of coronavirus fusion activation through study of SARS-Cov-2, in particular the H655Y mutation that is found in Omicron variants. The work nicely illustrates the complexities inherent to the evolution of the viral spike gene and how genomic changes play out in terms of altered function.

**Part II – Major Issues: Key Experiments Required for Acceptance**
Please use this section to detail the key new experiments or modifications of existing experiments that should be absolutely required to validate study conclusions.

Generally, there should be no more than 3 such required experiments or major modifications for a "Major Revision" recommendation. If more than 3 experiments are necessary to validate the study conclusions, then you are encouraged to recommend "Reject".

Reviewer #1: None

Reviewer #2: • Figures 3F and 3G: Statistical analysis is needed and should be included.

We have added these statistical analyses and modified the figure legends accordingly.

• Figures 5D and 5E: The differences between D614G and Omicron appear minimal in the plotted data despite p-values < 0.001. This discrepancy needs clarification. Additionally, p-values for Figure 5C should be reported.

Thanks, and yes, we also agree that the differences seen in the Fig 5D and 5E titration graphs are modest, with relatively large error bars. The error bars in the titration graphs on the left part of the Figs reflect the variabilities between ***technical*** replicates within a single experiment. Yet upon repeating the experiment 3 ***independent*** times, we found that relative IC50s fell quite close to each other, resulting in **statistically-significant** differences in IC50 across conditions. The error bars in the dot plots on the right depicts the variability of IC50s across three *biological* repeats. The high variability in the titration graphs reflect the low resolution of live-cell systems, which highlights the much higher resolution of our cell-free fusion system. This is stated now in line 229 of the updated manuscript. On a separate note, we have added the statistical analyses for Fig 5C and modified the figure legends accordingly.

Reviewer #3: 1) Much of the study hinges on differences between a previously reported neutralizing fusion-inhibitory MAb 4A8 (which recognizes the NTD) and a distinct MAb CC25.106 which recognizes the stem-helix. Using their VLP/EV system (which allows a degree of mechanistic specificity that would be difficult to obtain in cell-based assays), the authors do a good job in characterizing the differences between these two MAbs in terms of their action and the details of how the fusion reaction is inhibited, and link to a specific mutation, H655Y

Thank you.

2) As MAb CC25.106 is key to the present manuscript, but described in detail elsewhere (ref 57), the authors should better introduce this MAb for the current readers – eg epitope localization, cross-reactivity etc

Thank you for this important suggestion. In response, we have added a new panel in Fig 1 (Fig 1A) to schematically explain S structural components, the location of H655, and the location of stem-helix antibody epitope. We also elaborated the text describing the properties of stem-helix antibody on lines 94 and 290 of the updated manuscript.

3) Fig 1/2 are pretty narrow in terms of the range of SARS-CoV-2 species that are examined – this may be due to the limitations of assay used, but information of other VOCs, including a range of Omicrons and WA1/B.1 would be useful, especially as H655Y occurs in non-Omicron VOCs (eg Gamma)

Thank you for the suggestion. The main focus of the current manuscript is on deciphering functional S protein evolution from the ancestral D614G (aka B.1) to Omicron (aka BA.1 or 2). This allowed us to make new findings. We agree that characterizing the role of H655Y in other SARS-CoV-2 S backgrounds can be enlightening. We do try to stay up with continuous SARS-CoV-2 evolution beyond and BA.1 or 2. Along these lines, we are constructing more SARS-CoV-2 S variants, but these aims are currently in their early stages. We believe that our analyses of a range of Omicron variants will generate additional insights that are suitable for new manuscript. Therefore, in this submission, we channeled our efforts to decipher the initial evolution from the ancestral D614G to Omicron S. In future works, we will focus on the effects of subsequent adaptations within the Omicron subfamily.

4) The Discussion is well written and covers a lot of complex material on SARS-CoV-2 very well

Thank you.

**Part III – Minor Issues: Editorial and Data Presentation Modifications**
Please use this section for editorial suggestions as well as relatively minor modifications of existing data that would enhance clarity.

Reviewer #1: Although not critical for the paper, the authors should determine the IC50 of CC25.106 against newer Omicron variants, such as JN.1 or KP.3, which have the P681R substitution at the cleavage site, shown to improve fusogenicity.

Thank you for this comment. Indeed, there may be other adaptations that affects stem-helix antibody neutralization. As stated in our responses to reviewer #3, we agree that investigations of *recent* S evolution taking place within the Omicron-like variants can be very enlightening. As noted above, we feel that these new experiments deserve thorough consideration in a future manuscript. The current manuscript focuses on the initial evolution from the ancestral D614G to Omicron.

More importantly though, the authors should show the reversion of the H655Y phenotype in Omicron (mutating the Y back to a H). This is relevant for Figure 2 to show fusion blocking, Figure 4 to show the kinetics of fusion and Figure 5 to show this reversion in the context of live-cell virus entry assays. Does the reversion of this substitution result in even faster entry in the backbone of Omicron?

We have addressed this more important recommendation with new experiments and results. We reverted residue 655 in Omicron BA.1 (Y655H) and compared its phenotype to the parent BA.1 S. In agreement with our other results, the Y655H change reduced BA.1 S neutralization sensitivities towards HR2 peptides and stem-helix antibody CC25.106. We also answered the question about entry kinetics; the Y655H reversion resulted in even faster membrane fusion kinetics than Omicron BA.1. These data are consolidated into a newly-added supplemental figure 2 (Fig S2).

In Figure 3 it would be interesting to see pre-fusion S binding in the context of Omicron S and Omicron S with the H655Y substitution reverted.

Thank you for this comment. We have attempted, but unfortunately Omicron S appears to be more dynamic and could not survive the protocol for generating biotin-VLPs for VLP-antibody binding assay, which involves serial high-speed pelleting of VLPs.

Textually, the choice of “variations” in the title is confusing to me. The authors observed the effect of a single substitution, H655Y on antibody binding and S biochemistry during entry. Also in the abstract, what do the authors mean by “Omicron variations in the peripheral “S1” domains altered late-stage fusion dynamics”. They could just say instead that “peripheral S1 domains specific to Omicron altered late-stage fusion dynamics” or just focus on the single substitution of interest.

We understand that the findings did allow us to focus in on a substitution at residue 655, but this was not the “single substitution of interest”. We got to residue 655 only after analyzing many other substitutions. That is why we decided to broaden the title to “variations … reveal mechanisms…”. Also, in support of the broadened title, we do include findings showing that RBD substitutions also reveal mechanisms controlling virus entry dynamics. For these reasons, we want to keep the title as is. As for the abstract, we have changed the text in accordance with the reviewer’s suggestion. Thanks for the help with the abstract.

Figure 1, I suggest to split panel 1B into 1B and 1C.

We have split the old panel 1B into the new 1C and 1D.

To better follow the different domains of Spike and how they are affected, a diagram showing the Spike protein and all of its domains would be useful.

Thank you for this important suggestion. We agree this will significantly improve manuscript clarity. In response, we have added a new panel in Figure 1 (Fig 1A) to schematically highlight various S domains as well as inhibitor epitopes. We changed the text throughout the manuscript to indicate the new domain nomenclature.

Like in Figure 2D, similarly to showing at least whether reverting the H655Y substitution in the Omicron backbone would revert the phenotype, it would be interesting if the authors would look at all the substitutions added to the D614G backbone instead reverted in the Omicron backbone.

Thank you for this suggestion. In response, we have made the Y655H Omicron BA.1 revertant recombinant S construct. In agreement to the other data, Y655H revertant rendered BA.1 S less sensitive to HR2 and stem-helix antibody mediated neutralizations. Y655H also quickened the membrane fusion kinetics of BA.1 S. These data are compiled in the new supplemental figure 2 (Fig S2).

Please quantify the proteolytic cleavage in Figure 3C.

We have added the quantification of band intensity of the proteolytic product in Fig 3C.

Reviewer #2: • Omicron Lineages: Since Omicron has evolved into multiple lineages, the manuscript should specify the BA.1 lineage.

To increase clarity, we have added the designation “BA.1” in numerous locations throughout the manuscript.

• Terminology: The term "CoV" is used in many places to refer to SARS-CoV-2 and should be corrected for clarity.

Thank you for the comment. We have tightened, in several locations, our use of “SARS-CoV-2” for virus-specific interpretations, and “CoV” for statements that we believe will apply broadly to all CoVs.

Reviewer #3: Minor points, some of the wording in the text is not the best (eg “world dominance”, and what is meant by “opposition” in the abstract), are SD1 & 2 better defined as domains C & D?

We revised words in the text, as indicated in the marked-up copy. Importantly, in response to your suggestion to rename S domains, we did some major work to replace the former domain designations (NTD, RBD, SD1&2) to function-neutral nomenclature (S1A, S1B, S1C & D). We made updates throughout the manuscript to align this new nomenclature with the text and figures. We made these changes in response to this reviewer comment and also because the function-neutral nomenclature applies to all coronaviruses, particularly those with NTDs that function as “RBDs”. Thanks for prompting us to make these changes.

PLOS authors have the option to publish the peer review history of their article ([what does this mean?](https://nam12.safelinks.protection.outlook.com/?url=https%3A%2F%2Fc05y1x9s.r.us-east-2.awstrack.me%2FL0%2Fhttps%3A%252F%252Fjournals.plos.org%252Fplospathogens%252Fs%252Feditorial-and-peer-review-process%2523loc-peer-review-history%2F1%2F010f01920179c413-671e9458-6659-4ed1-9b8a-4eae889ea13e-000000%2FbgIDB0e9-NXw7YhmeSDEvhBnUvM%3D177&data=05%7C02%7Ceqing1%40luc.edu%7Cabbbd554d0d844a6df5808dcd75777d4%7C021f4fe32b9c48248378bbcf9ec5accb%7C0%7C0%7C638622017876516970%7CUnknown%7CTWFpbGZsb3d8eyJWIjoiMC4wLjAwMDAiLCJQIjoiV2luMzIiLCJBTiI6Ik1haWwiLCJXVCI6Mn0%3D%7C0%7C%7C%7C&sdata=OoW7uYcoOnAD1gWhBHEzDCHK3VFbOR7GmLdyM0t%2FA7Y%3D&reserved=0)). If published, this will include your full peer review and any attached files.


If you choose “no”, your identity will remain anonymous but your review may still be made public.


**Do you want your identity to be public for this peer review?** For information about this choice, including consent withdrawal, please see our [Privacy Policy](https://nam12.safelinks.protection.outlook.com/?url=https%3A%2F%2Fc05y1x9s.r.us-east-2.awstrack.me%2FL0%2Fhttps%3A%252F%252Fwww.plos.org%252Fprivacy-policy%2F1%2F010f01920179c413-671e9458-6659-4ed1-9b8a-4eae889ea13e-000000%2F3yy0E87MZlgyv8kimO_v_PrYLBk%3D177&data=05%7C02%7Ceqing1%40luc.edu%7Cabbbd554d0d844a6df5808dcd75777d4%7C021f4fe32b9c48248378bbcf9ec5accb%7C0%7C0%7C638622017876530756%7CUnknown%7CTWFpbGZsb3d8eyJWIjoiMC4wLjAwMDAiLCJQIjoiV2luMzIiLCJBTiI6Ik1haWwiLCJXVCI6Mn0%3D%7C0%7C%7C%7C&sdata=Re2pLnX9w9mZwmMY8HSIdHDELtSKy1UPu5ztz%2Bf%2FnN0%3D&reserved=0).

Reviewer #1: No

Reviewer #2: No

Reviewer #3: No

Figure Files:

While revising your submission, please upload your figure files to the Preflight Analysis and Conversion Engine (PACE) digital diagnostic tool, [https://pacev2.apexcovantage.com](https://pacev2.apexcovantage.com/). PACE helps ensure that figures meet PLOS requirements. To use PACE, you must first register as a user. Then, login and navigate to the UPLOAD tab, where you will find detailed instructions on how to use the tool. If you encounter any issues or have any questions when using PACE, please email us at figures@plos.org.

Data Requirements:

Please note that, as a condition of publication, PLOS' data policy requires that you make available all data used to draw the conclusions outlined in your manuscript. Data must be deposited in an appropriate repository, included within the body of the manuscript, or uploaded as supporting information. This includes all numerical values that were used to generate graphs, histograms etc.. For an example see here: [http://www.plosbiology.org/article/info%3Adoi%2F10.1371%2Fjournal.pbio.1001908#s5](https://nam12.safelinks.protection.outlook.com/?url=http%3A%2F%2Fc05y1x9s.r.us-east-2.awstrack.me%2FL0%2Fhttp%3A%252F%252Fwww.plosbiology.org%252Farticle%252Finfo%25253Adoi%25252F10.1371%25252Fjournal.pbio.1001908%2523s5%2F1%2F010f01920179c413-671e9458-6659-4ed1-9b8a-4eae889ea13e-000000%2FxAix0KqyGTxl2R__7oHgLbUoRwY%3D177&data=05%7C02%7Ceqing1%40luc.edu%7Cabbbd554d0d844a6df5808dcd75777d4%7C021f4fe32b9c48248378bbcf9ec5accb%7C0%7C0%7C638622017876538947%7CUnknown%7CTWFpbGZsb3d8eyJWIjoiMC4wLjAwMDAiLCJQIjoiV2luMzIiLCJBTiI6Ik1haWwiLCJXVCI6Mn0%3D%7C0%7C%7C%7C&sdata=KJKnwZEjR62xiZOa1%2BTST%2F%2FTJ2H2SIlI3Vt5jsWADgI%3D&reserved=0).

Reproducibility:

To enhance the reproducibility of your results, we recommend that you deposit your laboratory protocols in protocols.io, where a protocol can be assigned its own identifier (DOI) such that it can be cited independently in the future. Additionally, PLOS ONE offers an option to publish peer-reviewed clinical study protocols. Read more information on sharing protocols at [https://plos.org/protocols?utm_medium=editorial-email&utm_source=authorletters&utm_campaign=protocols](https://nam12.safelinks.protection.outlook.com/?url=https%3A%2F%2Fc05y1x9s.r.us-east-2.awstrack.me%2FL0%2Fhttps%3A%252F%252Fplos.org%252Fprotocols%253Futm_medium%3Deditorial-email%2526utm_source%3Dauthorletters%2526utm_campaign%3Dprotocols%2F1%2F010f01920179c413-671e9458-6659-4ed1-9b8a-4eae889ea13e-000000%2FZLQKHxdiuZht-sVq5Epl9-NWoGY%3D177&data=05%7C02%7Ceqing1%40luc.edu%7Cabbbd554d0d844a6df5808dcd75777d4%7C021f4fe32b9c48248378bbcf9ec5accb%7C0%7C0%7C638622017876547081%7CUnknown%7CTWFpbGZsb3d8eyJWIjoiMC4wLjAwMDAiLCJQIjoiV2luMzIiLCJBTiI6Ik1haWwiLCJXVCI6Mn0%3D%7C0%7C%7C%7C&sdata=A0FR6PAY2DF2%2BqoG%2Fu7vrBDI2z4rA784yDgzy2OXXQg%3D&reserved=0)

References:

Please review your reference list to ensure that it is complete and correct. If you have cited papers that have been retracted, please include the rationale for doing so in the manuscript text, or remove these references and replace them with relevant current references. Any changes to the reference list should be mentioned in the rebuttal letter that accompanies your revised manuscript. If you need to cite a retracted article, indicate the article’s retracted status in the References list and also include a citation and full reference for the retraction notice.

*In compliance with data protection regulations, you may request that we remove your personal registration details at any time.* [*(Remove my information/details)*](https://nam12.safelinks.protection.outlook.com/?url=https%3A%2F%2Fc05y1x9s.r.us-east-2.awstrack.me%2FL0%2Fhttps%3A%252F%252Fwww.editorialmanager.com%252Fppathogens%252Flogin.asp%253Fa%3Dr%2F1%2F010f01920179c413-671e9458-6659-4ed1-9b8a-4eae889ea13e-000000%2F4MuBCN_DSkQcXCAAvLCGiBZ63v8%3D177&data=05%7C02%7Ceqing1%40luc.edu%7Cabbbd554d0d844a6df5808dcd75777d4%7C021f4fe32b9c48248378bbcf9ec5accb%7C0%7C0%7C638622017876659514%7CUnknown%7CTWFpbGZsb3d8eyJWIjoiMC4wLjAwMDAiLCJQIjoiV2luMzIiLCJBTiI6Ik1haWwiLCJXVCI6Mn0%3D%7C0%7C%7C%7C&sdata=hayANZGRUheGG2dUfimichiUULuLwdkP27YR1ruWFo4%3D&reserved=0)*. Please contact the publication office if you have any questions.*
